# An Emergency Department Patient With Pharyngitis and Final Diagnosis of Rare T-cell Acute Lymphoblastic Leukemia With a Copy of Retinoic Acid Receptor Alpha Gene

**DOI:** 10.7759/cureus.39483

**Published:** 2023-05-25

**Authors:** Eman Shreteh, Susannah Boulet, Melody L Milliron, Christopher Karns

**Affiliations:** 1 Emergency Medicine, Lake Erie College of Osteopathic Medicine, Erie, USA; 2 Emergency Medicine, Allegheny Health Network - St. Vincent Hospital, Erie, USA

**Keywords:** oncology, retinoic acid receptor alpha gene, pharyngitis, hematology, t-cell acute lymphoblastic leukemia

## Abstract

A 21-year-old female with a past medical history of chronic tonsilitis presented to the emergency department (ED) with a sore throat and swelling in her neck for a two-week duration. The patient was noted to have pancytopenia with blasts on peripheral blood differential, so she was transferred for admission at an outside facility for further evaluation and management. Bone marrow biopsy revealed T-cell acute lymphoblastic leukemia (ALL) with 39.5% blasts. CALGB 10403 treatment protocol was initiated two days after her presentation to the ED. The patient also had an extra copy of the retinoic acid receptor alpha (RARA) gene. One year later, the patient was in remission, and cytogenetic results showed a normal female karyotype indicating that the patient no longer had ALL or RARA gene abnormalities.

While a sore throat can be a common chief complaint in the ED, ED providers need to keep a broad differential as there are many serious and potentially life-threatening etiologies such as T-cell ALL. T-cell ALL diagnosis is established with the presence of >20% of lymphoblasts in the bone marrow or peripheral blood draw. Cytogenetic changes play a significant role in determining the prognostic factors and management of ALL.

## Introduction

Acute lymphoblastic leukemia (ALL) is the second most common acute leukemia in adults, with an incidence in the United States of 1.8 per 100,000 in all age groups and five per 100,000 for ages 0-19 [1,2]. Maturational arrest during thymocyte development gives rise to T-cell ALL, which is triggered by multiple genetic aberrations. T-cell ALL is defined by 20% or more lymphoblasts in the bone marrow or peripheral blood [1,3]. Clinical manifestations of ALL reflect malignant, poorly differentiated lymphoid cells within the bone marrow, peripheral blood, and extramedullary sites [1]. In up to 20% of patients, these extramedullary changes cause splenomegaly, hepatomegaly, and lymphadenopathy [1]. Morphology, flow cytometry, cytogenetic testing, and immunophenotyping confirm the diagnosis and risk stratify the severity of illness. Other evaluations include assessing hematopoietic cell lines with complete blood count with differential and smear. In the evaluation for CNS involvement at the time of diagnosis, lumbar puncture with CSF analysis is standard.

## Case presentation

A 21-year-old female with a past medical history of chronic tonsillitis presented to the emergency department (ED) with two weeks of a sore throat and neck swelling. She also reported abdominal pain in the left upper quadrant on inspiration. She denied any fevers, chills, nasal congestion, rhinorrhea, cough, shortness of breath, chest pain, nausea, vomiting, change in bowel movement, or urinary symptoms. Physical examination revealed mild injection in the posterior pharynx and 2+ tonsillar hypertrophy bilaterally with no exudates or petechiae. The patient also had bilateral anterior and posterior cervical lymphadenopathy and left-sided preauricular lymphadenopathy. She had no axillary lymphadenopathy and was afebrile. Her rapid strep test was negative. Blood lab work showed significant leukocytosis of 65,300 with 27.4% blasts (Table 1). In addition, she had no signs or symptoms of any deep tissue infections such as Ludwig’s angina, retropharyngeal abscess, peritonsillar abscess, or bacterial tracheitis, all of which can also cause a sore throat with leukocytosis and lymphadenopathy. The patient was transferred for a higher level of care, and a bone marrow biopsy was performed. Her bone marrow biopsy and aspirate showed 39.4% blasts with a predominance of immature-appearing lymphoid cells consistent with lymphoid blasts. As T-cell ALL is defined by 20% or more lymphoblasts in the bone marrow or peripheral blood [1,3], this established a diagnosis of T-cell ALL.

**Table 1:** Lab results of our patient with pharyngitis symptoms and final diagnosis of ALL RARA, retinoic acid receptor alpha; ALL, acute lymphoblastic leukemia

Lab	Result
Rapid streptococcal antigen	Negative
CBC	WBC 65,300 with 27.4% blasts
Bone marrow biopsy and aspirate	39.4% blasts with a predominance of immature-appearing lymphoid cells consistent with lymphoid blasts, which is positive for CD3, CD4, CD8, CD2, CD5, CD7, CD99, and TDT
Flow cytometry	Demonstrated a surface CD3-, cytoplasmic CD3+, dual CD4+/CD8+, and TDT+ population consistent with T-lymphoid blasts, and MIB-1 for cell proliferation is also elevated
Cytogenetic studies	Revealed a duplication of the RARA gene

The patient underwent a treatment course following CALGB 10403 protocol with induction, consolidation, and maintenance phases. She was discharged on day 22. Bone marrow analysis was repeated and showed hypocellular marrow with no blastic infiltrates identified. Cytogenetic studies were repeated one year later, which demonstrated a normal female karyotype and was negative for the most common abnormalities found in ALL and retinoic acid receptor alpha (RARA) genes. The patient was found to be in remission.

## Discussion

Cytogenetic abnormalities involving chromosome structure and number, such as translocations and rearrangements, impact ALL prognosis [3,4]. The most common translocation found in ALL is the t(12;21) translocation. The Philadelphia chromosome (Ph) t(9;22) translocation, which creates BCR-ABL1 fusion, is associated with a poor prognosis [1]. The presence of t(9;22) in adult ALL can range from 15% to 50% and increases with aging [1]. Ph translocation can be seen in either B-cell ALL or T-cell ALL [3]. In adults, B-cell ALL accounts for 75% of cases; the remainder comprises T-cell ALL [1]. ALL is less common in adults than children, and the prognosis in adults is less favorable due to less genetic subtypes with favorable outcomes [5].

Although many patients with acute leukemia experience oral-pharyngeal symptoms [6], ALL may be easily overlooked during an initial presentation due to nonspecific symptoms such as fever, night sweats, fatigue, and shortness of breath. Signs of bone marrow failure may manifest with easy bruising, fatigue, dyspnea, and infections [1]. For this reason, it is important to keep a broad differential diagnosis when evaluating a patient in the ED.

Further testing of our patient revealed a diagnosis of T-cell ALL. Her bone marrow biopsy demonstrated 39.4% blasts with a predominance of lymphoid blasts, which is positive for CD3, CD4, CD8, CD2, CD5, CD7, CD99, and TDT. Flow cytometry demonstrated a surface CD3−, cytoplasmic CD3+, dual CD4+/CD8+, and TDT+ population consistent with T-lymphoid blasts. MIB-1 for cell proliferation was also extremely high.

Cytogenetic studies for this patient revealed a duplication of the RARA gene. Mutations involving the RARA gene cause acute promyelocytic leukemia (APL), which is characterized by an accumulation of atypical promyelocytes in the bone marrow and peripheral blood [7]. APL is a clinically distinct variant of acute myeloid leukemia (AML). APL presentation is related to complications of pancytopenia, infections, and hemorrhagic findings including menorrhagia, gingival bleeding, or epistaxis [3].

Treatment of APL is tretinoin (all-trans retinoic acid (ATRA)). None of APL clinical features or diagnostic cytology were present in this patient; therefore, ATRA treatment was not warranted. Our patient was treated for T-cell ALL via CALGB 10403 protocol, starting with the induction phase. Induction therapy includes vincristine, corticosteroids, and daunorubicin, followed by PEG-asparaginase a week later. The treatment course for the patient continued through course V based on the CALGB protocol. She was maintained on allopurinol and was monitored for tumor lysis syndrome by obtaining baseline lactate dehydrogenase.

## Conclusions

ALL is a malignant proliferation and differentiation of lymphoid progenitor cells in the blood, bone marrow, and extramedullary sites. Definitive diagnosis is established with the presence of >20% of lymphoblasts in the bone marrow or peripheral blood. Cytogenetic changes play a significant role in determining the prognostic factors and management of ALL. Chemotherapy consists of induction, consolidation, and maintenance. Evaluating patients during treatment is accomplished by obtaining flow cytometry and PCR to assess minimal residual disease (MRD).

Clinical manifestations of ALL reveal an accumulation of the poorly differentiated cells. A physician may easily overlook ALL during an initial presentation due to symptoms such as sore throat, fever, night sweats, fatigue, and shortness of breath. Patients may also manifest extramedullary symptoms such as splenomegaly. Keeping a broad differential is key to a more rapid diagnosis, which can potentially improve the patient’s outcome by more rapid treatment onset.
